# Early specification of dopaminergic phenotype during ES cell differentiation

**DOI:** 10.1186/1471-213X-7-86

**Published:** 2007-07-18

**Authors:** Malin Parmar, Meng Li

**Affiliations:** 1 Institute for Stem Cell Research, University of Edinburgh, Edinburgh, UK; 2 MRC Clinical Sciences Centre, Faculty of Medicine, Imperial College, London, UK

## Abstract

**Background:**

Understanding how lineage choices are made during embryonic stem (ES) cell differentiation is critical for harnessing strategies for controlled production of therapeutic somatic cell types for cell transplantation and pharmaceutical drug screens. The in vitro generation of dopaminergic neurons, the type of cells lost in Parkinson's disease patients' brains, requires the inductive molecules sonic hedgehog and FGF8, or an unknown stromal cell derived inducing activity (SDIA). However, the exact identity of the responding cells and the timing of inductive activity that specify a dopaminergic fate in neural stem/progenitors still remain elusive.

**Results:**

Using ES cells carrying a neuroepithelial cell specific vital reporter (*Sox1*-GFP) and FACS purification of *Sox1*-GFP neural progenitors, we have investigated the temporal aspect of SDIA mediated dopaminergic neuron specification during ES cell differentiation. Our results establish that SDIA induces a dopaminergic neuron fate in nascent neural stem or progenitor cells at, or prior to, *Sox1 *expression and does not appear to have further instructive role or neurotrophic activity during late neuronal differentiation of neural precursors. Furthermore, we show that dopaminergic neurons could be produced efficiently in a monolayer differentiation paradigm independent of SDIA activity or exogenous signalling molecules. In this case, the competence for dopaminergic neuron differentiation is also established at the level of *Sox1 *expression.

**Conclusion:**

Dopaminergic neurons are specified early during mouse ES cell differentiation. The subtype specification seems to be tightly linked with the acquisition of a pan neuroectoderm fate.

## Background

Understanding how lineage progenitors are specified to give rise to distinct somatic cell types is fundamental for devising strategies for controlled differentiation of stem cells. It is generally believed that during in vitro differentiation, embryonic stem (ES) cell-derived neural stem cell/progenitors acquire a dorsal-ventral and anterior-posterior regional identity in a similar fashion to that of neuroepithelial stem cells in the developing embryo. Distinct regional identity, as demonstrated by the expression of a range of transcription factors, could be established by developmental key morphogens such as sonic hedgehog (SHH), bone morphogenic proteins (BMPs), and retinoic acid (RA) [1-4]. For example, SHH and FGF8 have been wildly used as inducers for generating dopaminergic neurons whilst RA is employed to direct motorneuron production from ES cells [2,4-6]. These inductive molecules are generally applied at the time when neural progenitor production (ie. nestin^+^, Sox1^+ ^cells) is at its peak. These studies might imply that the morphogens such as Shh and FGF8 act on ES cell-derived nestin^+^, Sox1^+ ^neural progenitors to impose a regional identity. However, due to the heterogeneous nature of ES cell in vitro differentiation, one can not firmly pin point the identity of the responsive cells. Furthermore, neural fate acquisition from ES cells in vitro can occur much more quickly than in vivo during development [7-9], implying that patterning during ES cell differentiation may not fully recapitulate embryo development.

In addition to Shh and FGF8, unknown inductive molecules that are produced by stromal or other cell lines have also been exploited to direct lineage specific differentiation [10-12]. For example, dopaminergic neurons can be induced by co-culturing ES cells with bone marrow derived stromal cells such as PA6 that exhibit stromal cell-derived inducing activity (SDIA; [10]). However, at which stage of ES cell differentiation SDIA acts, and to what extent regional identity is acquired intrinsically in this model system and other ES cell differentiation paradigms remains largely unknown.

We have previously developed an ES cell model system that allows visualization and identification of neural stem cells/progenitors during ES cell differentiation by knocking in a GFP reporter into the *Sox1 *locus [13]. During ES cell differentiation, *Sox1*-GFP is detected earlier than nestin in ES cell-derived neural progenitors [14], thus providing a valuable tool to purify these cells from undifferentiated ES cells and non-neural cell types by fluorescence activated cell sorting (FACS). In this study, we utilize the *Sox1*-GFP model system in combination with either PA6 co-culture or monolayer ES cell neuronal differentiation in order to investigate the temporal aspects of dopaminergic specification during ES cell differentiation.

We provide evidence that SDIA promotes dopaminergic fate specification in neural progenitors at or prior to *Sox1 *expression and that SDIA does not appear to have further instructive role or neurotrophic activity during neuronal differentiation of neural precursors. Furthermore, our work suggests that dopaminergic specification can occur efficiently independent of SDIA activity and similarly at the level of *Sox1 *expression, suggesting that early specification is a general feature of dopaminergic differentiation from mouse ES cells.

## Results

### Dopaminergic specification occurs early during ES cell differentiation

**Figure 1:**
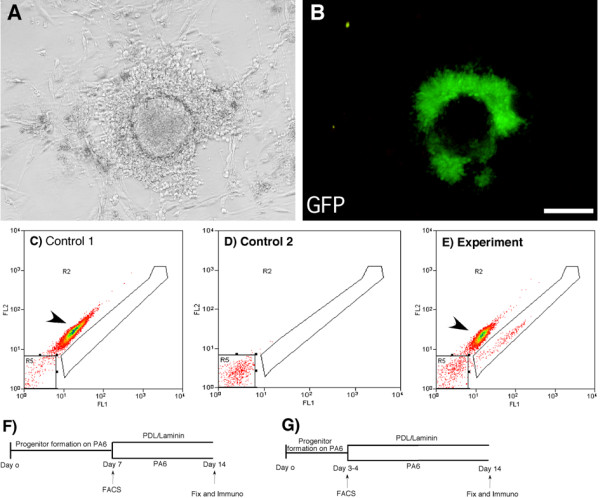
**FACS sorting of PA6-derived *Sox1*-GFP^pos ^neural progenitors**. Neural progenitors generated by co-culturing ES cells with PA6 can be identified based on GFP reporter expression from the *Sox1 *locus (A, B). FACS plots showing the isolation of GFP^pos ^neural progenitors from GFP^neg ^ES cell differentiated progeny and PA6 cells (C – E). Gates were set based on plots of PA6 cells + wild type ES cells (C) and wild type ES cells alone (D). FACS sorting was performed either on day 7 (F) or day 3–4 (G). *Sox1*-GFP^pos ^cells and GFP^neg ^differentiated ES cell population were subsequently cultured under neuronal differentiation conditions either on PDL/laminin or in co-culture with PA6 cells. After a total of 14 days in cultures, cells were fixed and processed for immunocytochemistry. FL1 = GFP, FL2 = PE, R2 = GFP^pos ^neural progenitors, R5 = GFP^neg ^differentiated ES cells. Arrowhead points PA6-enriched cell population. Scale bar = 100 μm.

**Figure 2:**
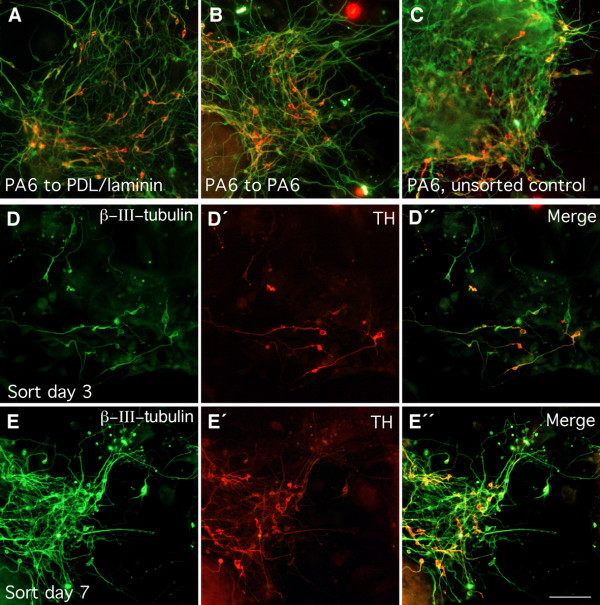
**Generation of TH^+ ^neurons by purified *Sox1*-GFP expressing progenitors**. Neural progenitors generated by co-culture with PA6 were FACS purified at day 3 or day 7. Following re-plating and differentiation, cultures were fixed and double stained with antibody against β-III-tubulin (green) and TH (red). A similar number of TH expressing neurons was produced by PA6-derived progenitors after being re-plated on PA6 (A) or PDL/Laminin (B) as compared to unsorted controls (C). *Sox1*-GFP expressing neural progenitors purified at day 3–4 (D, D', D") generated a similar proportion of TH expressing neurons as compared to those isolated at day 7 (E, E', E"). Scale bar = 100 μm.

In order to address the question whether SDIA acts on neural progenitors before or after the expression of *Sox1*, we exploited the *Sox1*-GFP reporter ES cells in combination with neural differentiation via co-culture with PA6 stromal cells. *Sox1*-GFP expressing neural progenitor cells were generated and purified by FACS sorting (Fig 1A–B, E). Most experiments were performed at day 7 of differentiation when the numbers of GFP expressing cells peaks (59 ± 6%). FACS sorted GFP^pos ^and GFP^neg ^cell populations, together with reconstituted mixed cultures as control cells, were collected and re-plated in parallel. The cells were plated either back on a layer of PA6 stromal cells or on PDL/laminin coated plastic in N2/B27, in the absence of exogenous growth factors (See flow scheme Fig. 1F, G). Following an additional 7 days of differentiation, cultures were examined for the presence of TH expressing neurons by immunocytochemical staining (Fig. 2A–C).

**Figure 3:**
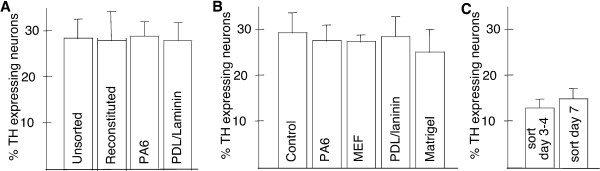
**Quantification of TH expressing neurons derived from PA6-formed progenitors**. *Sox1*-GFP expressing neural progenitors generated by co-culture with PA6 sorted at day 3–4 or day 7 and allowed to differentiate for a further 7 days under various conditions. No difference was observed in the number of neurons that expressed TH in unsorted control cultures, reconstituted cultures, sorted GFP^pos ^cells re-placed onto PA6 or onto PDL-Laminin coated plastic (A) or when plated back onto different coatings or feeder cells (B). Likewise, no difference was found in the generation of TH expressing neurons between neural progenitors sorted at day 3–4 and day 7(C).

When the GFP^pos ^and GFP^neg ^populations were mixed again after sorting and plated back onto PA6 cells, they gave rise to TH positive neurons at a frequency of 27 ± 7%. This frequency is indistinguishable from that of unsorted parallel control cultures (28 ± 4%, Fig. 3A), verifying that the sorting procedure itself had no adverse effect on subsequent dopaminergic differentiation. Therefore, we used unsorted cultures as controls in subsequent experiments. The GFP^neg ^population, which consists mostly of cells of non-neural lineage and a minority of undifferentiated ES cells, rarely give rise to TH expressing neurons in the subsequent 7 day differentiation culture regardless of the re-plating condition after sorting (data not shown), consistent with previously reported kinetics of dopaminergic neuron differentiation [10,15]. Interestingly, we obtained a similar proportion of TH expressing neurons from FACS purified *Sox1*-GFP expressing neural progenitors as compared to unsorted culture after re-plating on PA6 cells or on PDL/laminin (Fig. 2A–C, Fig 3A), suggesting that SDIA acts early during ES cell differentiation and that neural progenitors in this differentiation paradigm are already patterned to generate TH expressing neurons before or at the time of *Sox1 *expression.

Since the production of neural progenitors during ES cell in vitro differentiation is not synchronized, some of the FACS purified cells might have been produced at an earlier time point which have already committed to neuronal differentiation and lost the competence to respond to SDIA despite the maintenance of GFP expression. We have therefore examined the early born neural progenitors by performing FACS sorting at day 3–4 after plating. This is the earliest time point when *Sox1*-GFP, nestin, and Sox1 expressing cells can be readily detected. We found that day 3–4 *Sox1*-GFP^pos ^neural progenitors produced a similar proportion of TH^+ ^neurons as compared to day 7 neural progenitors. (Fig 2D–E, 3C). Thus this data further supports the notion that the competence to generate TH expressing neurons by neural progenitors is closely linked to the onset of Sox1 expression.

### Early specification of dopaminergic competent neural progenitors in the absence of SDIA or exogenous inductive molecules

**Figure 4:**
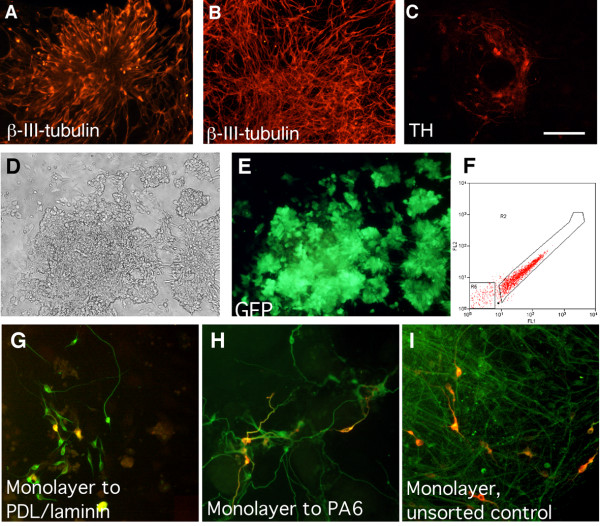
**Generation of TH^+ ^neurons via monolayer differentiation**. A substantial number of neurons start to appear after 10 days of monolayer differentiation (A). After 14 days of differentiation, more mature neurons can be detected (B) and some also express TH expressing (C). Scale bar = 100 μm. Monolayer-derived neural progenitors were identified and isolated based on GFP expression (D-F). TH expressing neurons were generated after a total of 14 days of differentiation when cells were plated on PDL/laminin (G), in co-culture with PA6 cells (H) after sorting as well as in control cultures (I).

**Figure 5:**
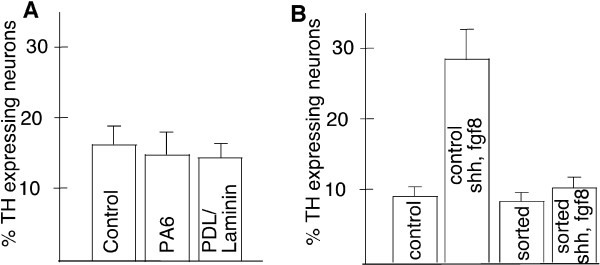
**Quantification of TH expressing neurons derived from monolayer-formed progenitors**. *Sox1*-GFP expressing neural progenitors were sorted from day 7 monolayer cultures and allowed to differentiate for a further 7 days under various conditions. No differences was found in the proportion of TH expressing cells generated by unsorted control cultures, sorted GFP^pos ^neural progenitors re-placed on PA6 or PDL-Laminin coated plastic (A). In unsorted control cultures, the number of TH expressing neurons increased when SHH/FGF8 was applied during differentiation. However, addition of SHH/FGF8 to purified *Sox1*-GFP cells had no effect on the number of TH expressing neurons produced.

To investigate whether the observed early dopaminergic fate specification in *Sox1 *expressing neural progenitor cells reflects a general mechanism or is specific for the PA6 differentiation method, we examined dopaminergic neuron production in another differentiation paradigm: monolayer differentiation. In contrast to previous reports where few dopaminergic neurons are generated in the absence of Shh and FGF8 [9,18], we found that a substantial number of TH expressing cells are consistently and robustly produced without feeder cells or the addition of extrinsic factors during standard monolayer differentiation in N2/B27 on gelatin-coated plastic (Fig 4 and 5). Under these conditions, very few, if any, TH expressing cells were present at 10 days of differentiation although many β-III-tubulin expressing neurons can be detected at this time (Fig. 4A). After another 4 days of differentiation (a total of 14 days), more neurons (Fig 4B) and many TH expressing cells can be detected (Fig. 4C), corresponding to 16 ± 3% of the number of β-III-tubulin expressing neurons. The TH expressing cells were frequently observed in clusters and all had the morphology of neurons (Fig. 4C). 91 ± 7% of these TH expressing neurons also expressed Nurr1.

To address whether the dopaminergic fate specification also occurred already in the *Sox1*-GFP expressing neural progenitors produced in monolayer differentiation, GFP expressing cells were sorted using the same criteria as described for PA6 cultures and re-plated on PDL/Laminin coated plastics in N2B27 medium. Cell sorting was performed after 7 days when 81 ± 9% of the cells expressed GFP (Fig. 4D–F). Unsorted cultures processed in parallel gave rise to TH expressing neurons at a frequency of 16 ± 3% (Fig. 4I, 5A). Again, re-mixed FACS purified GFP^pos ^and GFP^neg ^populations produced a similar number of TH expressing neurons to the unsorted controls (not shown). The *Sox1*-GFP expressing population gave rise to TH expressing neurons (Fig. 4G–I) at a frequency indistinguishable from that of unsorted controls (14 ± 2% versus 16 ± 3%) after being re-plated on PDL/laminin (Fig. 5A), suggesting that the ability to differentiate into TH expressing neurons by monolayer-derived progenitors is also specified at the level of *Sox1*-GFP expression.

### PA6/SDIA, but not monolayer differentiation, promotes formation of midbrain-like dopaminergic neuron progenitors and neurons

Midbrain property is key for functional integration of transplanted dopaminergic neurons [19]. Previous studies suggest that ES cell derived dopaminergic neurons are heterogeneous with regard to regional identities and only a proportion of ES cell-derived TH^+ ^neurons are characteristic of midbrain dopaminergic neurons [3,6,15,18,20]. Therefore we examined the acquisition of midbrain dopaminergic progenitor fate and its relation to *Sox1*-GFP expression during neuronal differentiation by co-culture with PA6 stromal cells or on PDL/laminin.

We first validated the purity of FACS sorted *Sox1*-GFP neural progenitors for contamination of undifferentiated ES cells using antibodies against Oct4 and Sox2. Very few (less than 5%) of the *Sox1*-GFP^pos ^cells, derived either from PA6 co-culture or from monolayer differentiation, co-expressed Oct4 (Additional file 1). As expected and similar to neural progenitors residing in the proliferative zone of the developing midbrain, more than 90% of the *Sox1*-GFP expressing cells also expressed Sox2 (Additional file 1).

**Figure 6:**
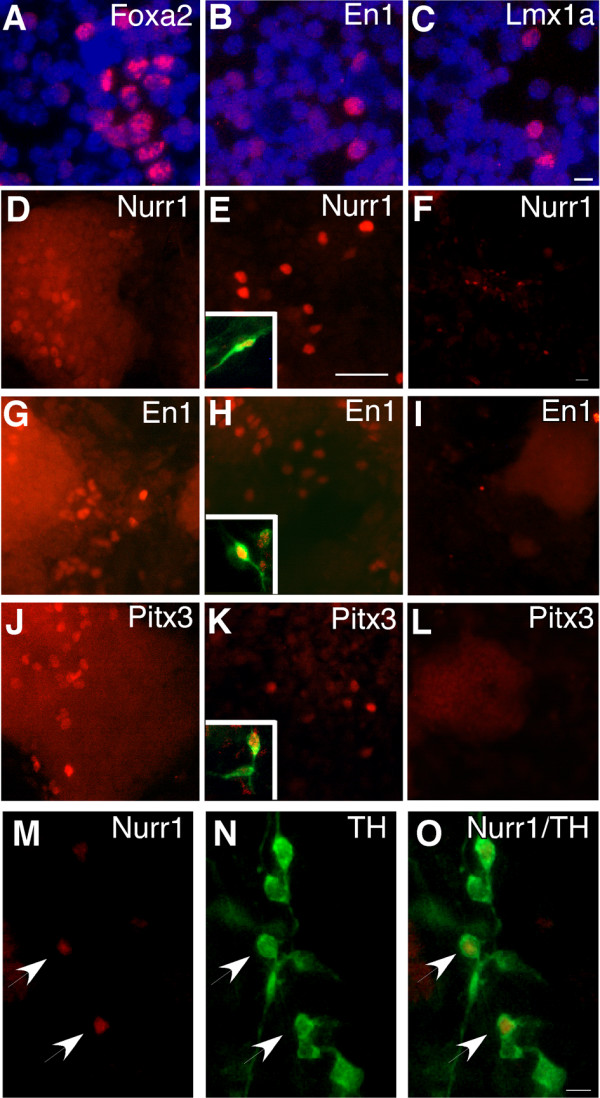
**Expression of midbrain markers by *Sox1*-GFP^+ ^neural progenitors and their differentiated progeny**. *Sox1*-GFP^pos ^neural progenitors, generated by co-culture with PA6 (A-C, D, E, G, H, J, K) or from monolayer culture (F, I, L-O), were purified by FACS. Cells were either collected onto slides by cytospin (A-C) or re-plated on PA6 or PDL/laminin and allowed for differentiation for a further 7 days (D-O). Cells collected on slides were examined for the expression of midbrain dopaminergic progenitor markers by immunostaining using antibodies against FoxA2 (A), En1 (B) and Lmx1a (C). Cultures were processed for the expression of midbrain dopaminergic neuron markers including Nurr1 (D-F), En1 (G-I), and Pitx3 (J-L) in conjunction with TH (inset E, H, K). Nurr1, En1 and Pitx3 was detected in cells derived from PA6 formed progenitors (D, E, G, H, J, K) whereas only Nurr1 (F, M-O) was found to be expressed in cells derived from monolayer-derived neural progenitors. Scalebar for A-C and M-O = 10 μm, scalebar for D, E, G, H, J, K = 50 μm and scalebar for F, I, L = 50 μm.

We found that a proportion of the PA6 co-culture derived neural progenitors express midbrain markers including FoxA2 (Fig. 6A), En1 (Fig. 6B), and Lmx1a (Fig. 6C). In keeping with developmental studies, postmitotic midbrain DA precursor markers such as Nurr1 and Pitx3 were not detected in the *Sox1*-GFP^pos ^population. However, after an additional 7 days of neuronal differentiation, either on PDL/Laminin (Fig. 6E,H,K) or maintained in contact with PA6 (Fig. 6D,G,J), these *Sox1*-GFP^pos ^progenitors gave rise to cells that expressed Nurr1, Pitx3, and En1 (Fig. 6D,E,G,H,J,K). Pitx3 expressing cells always co-expressed TH (inset in Fig 6E). Nurr1 and En1 were often, but not always, co-expressed with TH (Inset in Fig 6H and 6K). In PA6-derived cultures, the proportion of TH expressing cells that also expressed Nurr 1, En1 or Pitx3 was similar regardless of subsequent differentiation condition after FACS sorting: 82% (PA6-differentiated, n = 102) and 86% (PDL/laminin-differentiated, n = 111) of the TH expressing cells also expressed Nurr1, 20% (PA6-differentiated, n = 98) and 24% (PDL/laminin-differentiated, n = 103) co-expressed En1, and 16% (PA6-differerntiated, n = 100) and 14% (PDL/Laminin-differentiated, n = 99) co-expressed Pitx3.

Similar to the PA6 derived progenitors, *Sox1*-GFP^pos ^progenitors obtained by monolayer differentiation co-expressed Sox2 but not Oct4 (Supplemental Fig. 1). The majority of monolayer-derived TH expressing neurons, either produced from FACS purified *Sox1*-GFP progenitors re-plated on PA6 cells or directly from unsorted control cultures, expressed post mitotic dopaminergic neuron precursor marker Nurr1 (Fig. 6F, M–O). However, very few, if any, displayed a midbrain character at the level of *Sox1*-progenitor stage as determined by the lack of expression of FoxA2, En1, and Lmx1a. Furthermore, markers normally expressed by midbrain dopaminergic neurons such as En1 and Pitx3 or by diencephalic or olfactory bulb dopamine neurons such as Pax6, Nkx2.1, or Gad67 could not be detected in monolayer derived TH expressing neurons under any differentiation conditions tested (Fig. 6I,L and data not shown). Thus, our data demonstrate that neural progenitors produced from PA6 co-cultures can differentiate into dopaminergic neurons with midbrain characteristics, whilst monolayer differentiation paradigm does not support the acquisition of a midbrain dopaminergic neuron fate.

### SDIA does not appear to have further instructive role or neurotrophic activity during neuronal differentiation

We noticed that the GFP^pos ^cells plated on PDL/laminin or matrigel coated plastic in the absence of PA6 cells gave rise to TH expressing neurons at the same frequency as those re-plated on PA6 (Fig. 3B). Furthermore, embryonic mouse fibroblast cells (MEFs), which also exhibit some SDIA activity [10], did not have an effect on the production of TH^+ ^neurons (Fig. 3B). Therefore, our data suggest that SDIA does not promote terminal dopaminergic neuronal differentiation. To further address this question, we compared TH^+ ^neuron production of FACS purified monolayer derived *Sox1*-GFP progenitors re-plated either in co-culture with PA6 cells or alone on PDL/laminin. We found that a similar numbers of TH^+ ^neurons were generated under both culture conditions independent of the presence of PA6 cells at later stages of differentiation (Fig. 4G,H). 14 ± 2% of the neurons expressed TH when *Sox1*-GFP progenitors were re-plated on PDL/Laminin (Fig. 5A), whilst a similar level (15 ± 3%) was obtained when progenitors were re-plated in co-culture with PA6 cells (Fig. 5A). Thus, while the overall frequency of dopaminergic neuronal production by monolayer-derived neural progenitors is significantly (p ≤ 0.05) lower than that of PA6-formed progenitors, the exposure to SDIA during later stages of neuronal differentiation has no effect on the production of TH^+ ^neurons. Similar results were obtained when monolayer derived *Sox1*-GFP expressing progenitors were sorted on day 3 of ES cell differentiation and then plated on PA6 cells for the remainder of the differentiation period. Taken together, our data showed that, SDIA does not promote dopaminergic neuron differentiation or maturation after *Sox1*-GFP expression.

To address whether monolayer-formed *Sox1*-GFP^pos ^neural progenitors are able to respond to SHH and FGF8, molecules well known to promote dopaminergic neuron production in both mouse and human ES cells, we added SHH and FGF8, together with FGF2 to cultures after FACS sorting of monolayer-formed progenitors at concentrations previously described to promote dopaminergic specification during ES cell differentiation [9,21]. While continuous differentiation cultures under the same treatment produced significantly more TH^+ ^neurons (Fig. 5B, and as reported previously [2,6,11,15], no increase in TH expressing cells was observed from FACS purified neural progenitors (Fig. 5B) and no midbrain dopaminergic marker expression was induced within the TH expressing population. Therefore, our results suggest that the competence for dopamine neuron differentiation is acquired very early on during mouse ES cell in vitro development and is in-separable from the establishment of a pan-neuroectodermal fate.

## Discussion

Restorative cell therapy for Parkinson's disease, where immature dopaminergic neurons are isolated from early gestation foetuses and transplanted into the striatum of PD patients, has been used with promising results [22]. However, to carry this approach forward and to circumvent ethical and practical issues associated with using primary foetal cells, an alternative strategy for obtaining unlimited numbers of cells for grafting is essential. ES cells constitute a promising source of such cells, but much remains to be elucidated about how neurons derived from ES cells are patterned as well as how and when they acquire an anterior-posterior and dorsal-ventral identity that allows them to differentiate into neurons with specific characteristics, such as midbrain dopaminergic neurons.

Studies using primary cell grafts have shown that survival of dopamine neurons in the host brain decreases with developmental age of the donor tissue [23], suggesting that when grafting dopamine neurons in PD models it is important to use immature cells that survive the procedure well. It is also essential that the cells grafted have been sufficiently patterned so that they are capable of differentiation into fully mature midbrain dopaminergic neurons in the host brain. Thus, in order to use ES cells derived neuronal precursors in transplantation experiments, it is important to determine at what stage the cells are sufficiently patterned to generate the correct phenotype in the host brain, but yet immature enough to survive the grafting procedure.

Co-culturing with PA6 cells has been reported to be an effective method for generating neurons from both mouse and human ES cells [10,15,24,25]. Differentiation of SDIA-treated ES cells *in vitro *appears to mimic the natural course of *in vivo *neurogenesis as judged by the temporal expression of neural progenitor and differentiated neuronal markers [10]. No significant amount of mesodermal cells are formed in this differentiation paradigm, and the neuronal subtypes generated include that of forebrain, midbrain and hindbrain although cells with characteristics of spinal cord neurons has not been reported [10,26]. Of particular interest is that a significant portion of the neurons generated express TH and a sub-portion also express other markers characteristic of midbrain neurons [10,15]. The PA6-derived neurons produce and release dopamine and have been shown to functionally integrate after intracerebral grafting [10,27]. *Sox1*-GFP expressing cells purified from PA6 cultures also differentiate into dopamine neurons after transplantation [16,17].

By utilizing the *Sox1*-GFP model system, where GFP faithfully mimics the expression of endogenous Sox1 in the entire developing neuroepithelium during early developmental stages and in neural progenitors *in vitro *[9,14,28], we were able to determine at what stage SDIA acts during ES cell differentiation, and to what extent regional identity is acquired intrinsically in this model system as well as in other ES cell differentiation paradigms. Sox1 is expressed in all neural progenitor cells at early developmental time points but is cleared from the ventral midbrain by the time dopamine neurons are generated during development [13,28]. Therefore, whether midbrain dopaminergic progenitors are derived from Sox1 expressing cells remains unknown. Our study shows directly that TH expressing neurons are indeed derived from the *Sox1*-GFP^pos ^population, providing further support of using ES cell model for lineage analysis [29,30]. The notion that midbrain progenitors are part of the *Sox1*-GFP^pos ^population is further supported by the fact that they also express Sox2, En1, Lmx1a, and FoxA2.

Furthermore, we show unambiguously that SDIA acts at an early stage of ES cell differentiation, most likely at the level of neural progenitor formation since the cells are already specified at the *Sox1 *expressing neural progenitor stage. This conclusion is supported by data obtained with both early born (day 3–4) and late born (day 7) *Sox1*-GFP^pos ^neural progenitors. During ES cell differentiation *Sox1*-GFP expression precedes that of nestin [14], lending further support to our notion. After the neural progenitor stage, SDIA can no longer direct the formation of dopaminergic neurons or act as a survival factor during neuronal differentiation. Early specification of ES cell-derived neural progenitors also seems to be the case for telencephalic fates, as the first 5 days of the induction period is decisive in telencephalic specification during ES cell differentiation in a similar, but feeder free, culture system [26]. It is interesting to note that this early specification seems to be a general feature of dopaminergic differentiation from ES cells, as we obtained the same result in a monolayer culture system that is not dependent on feeder cells or the addition of extrinsic patterning molecules. Thus, fate specification and progenitor formation may occur concurrently and may be instructed by the same signals, suggesting that neuronal specification and fate determination during ES cell differentiation are two closely linked processes that may not always be uncoupled. This phenomenon is to some extent different from neural progenitors in the embryo since an alternative regional identity of neural tissues can be induced by morphogenes when applied within a certain developmental time window [31].

However, it would be interesting to investigate whether a similar fast track fate specification also occur in human ES cells where the generation of any given cell type takes much longer time than the mouse counterpart. In this regard it is worth mentioning that ES cell-derived neural progenitors appears to have a wider time window of competence to adopt posterior cell fates, such as spinal cord identity [32], which is normally formed during a relatively longer period of time during development.

The current study also presents the first report on robust production of dopaminergic neurons using a monolayer differentiation protocol without the need for feeder cells or addition of extrinsic patterning molecules such as Shh and Fgf8. Monolayer-derived TH expressing neurons co-express Nurr1, a gene marker that is expressed in dopaminergic neurons but its expression is excluded from other catecholaminergic neurons [33]. Thus the TH^+ ^neurons generated are likely dopaminergic. However, these cells do not express any additional markers characteristic of a mesencephalic phenotype.

Approximately 25% of the dopaminergic neurons in the central nervous system are located outside the midbrain. Instead of En1 and Pitx3, these non-midbrain dopaminergic neurons express other proteins characteristic of their stereotypic position (reviewed in [34]). For example, diencephalic dopamine neurons express Pax6, Dlx, and Nkx2.1 and the dopaminergic neurons in the olfactory bulb are GABA-ergic and also express Pax6 and Dlx [3,35-37]. No Nkx2.1, Pax6 or Gad67 (marking GABA-ergic neurons) expression was detected in our monolayer culture derived TH expressing neurons. Thus, these neurons do not seem to bare characteristics for any specific dopaminergic subtype. The lack of specific regional characteristics might be due to limited inductive signals produced in monolayer differentiation conditions. In this regard, it is worth mentioning that SHH and FGF do not appear to have dopaminergic neuron promoting activity in purified Sox1^pos ^neural progenitors, despite the fact that these molecules induce dopaminergic neuron production in heterogeneous ES cell differentiation culture [21,31,38]. Thus these inductive molecules either only work during a narrow time window prior to or at the point of Sox1 expression during ES cell development and/or act in collaboration with unknown co-factors produced by non-neural cells.

## Conclusion

Detailed knowledge of the effects and timing of patterning signals are essential for planning prospective strategies to produce specific neuronal subtypes from ES cells. We show that dopaminergic specification occurs early during mouse ES cell differentiation and is tightly linked with the acquisition of a pan neural fate. Our results suggest that the time-window for dopaminergic specification is quite narrow and that attempts to increase the number of dopaminergic neurons should be focused on the early neural induction stage during ES cell in vitro differentiation than at later stages of neuronal differentiation.

## Methods

### Cell culture

*Sox1*-GFP ES cells were maintained in Glasgow Modified Eagles Medium (GMEM) supplemented with 2-mercaptoethanol, nonessential amino acids, sodium bicarbonate, 10% fetal calf serum and leukemia inhibitory factor (LIF) in gelatinized tissue culture flasks. Differentiation on PA6 stromal cells was carried out as previously described [10]. Briefly, ES cells were cultured on a layer of PA6 stromal cells for 7 days in GMEM supplemented with 10% knock-out serum replacement (Gibco) at a density of 60 cells/cm^2^. At day 7 medium was replaced with N2B27 (StemCellSciences) for the remainder of the differentiation period.

Monolayer differentiation was carried out as previously described [8,9]. Briefly, ES cells were plated on gelatinized tissue culture plastics in N2B27 at a density of 10 000 cells/cm^2^. Both types of differentiation cultures were processed for FACS either at day 3–4 or 7 followed by for immunostaining at day 14.

### FACS sorting

Cells were trypsinized and resuspended in 1% BSA in phosphate buffered saline (PBS) and filtered using a cell strainer (BD Falcon) to ensure single cell suspension. All cell sorting experiments were performed on a MoFlo cell sorter (DAKO). Live cells were gated based on forward scatter and side scatter and/or by 7AAD dye exclusion. Where applicable, the majority of the PA6 feeder cells were excluded based on their differential appearance from that of ES cell derivatives on PE (autoflouresence)/FITC plot. Gates for the two cell populations were set using PA6 cells and differentiated parental E14TG2a ES cells (Figure 1C, D). In some experiments, PA6 cells were excluded based on their inability to exclude 7-AAD. The GFP^pos ^neural progenitor population and the GFP^neg ^differentiated ES cell fraction were collected and cell viability at the end of the FACS sorting procedure was determined using trypan blue dye exclusion method. FACS sorted cells were either plated for neuronal differentiation or directly processed for cytospin.

### Neuronal differentiation

FACS sorted cells were re-plated either on Poly-D-lysine(PDL)/Laminin coated plastics or on PA6 stromal cells, at a density of 100 000 cells/cm^2 ^in both cases. Where indicated SHH (400 ng/ml, R&D), FGF8 (100 ng/ml, R&D), and FGF2 (10 ng/ml. R&D) was added to the N2B27 medium. At the end of the culture period, cells were fixed in ice cold 4% paraformaldehyde (PFA) for 15 min at room temperature followed by 3 rinses in PBS.

### Cytospin

FACS sorted cells were diluted to a concentration of 1 × 10^5 ^cells/ml. 100 μl of cell suspensions of each sample were spun at 1000 rpm for 4 minutes. Cells were immediately fixed in 4% PFA for 15 minutes at room temperature followed by 3 rinses in PBS.

### Immunostaining

Fixed cells were blocked with 5% normal serum and 0.2% triton X-100 for 1 hour followed by incubation overnight with primary antibodies: rabbit anti-TH (1:1000, Pel Freeze), mouse anti-TH (1:1000, chemicon or 1:500, PelFreeze), mouse anti-β-III-tubulin (1:500, Babco), rabbit anti-Pitx3 (1:500, gift of Dr. M Smidt), mouse anti-En1 (1:250, DSHB), rabbit anti-Nurr1 (1:500, SantaCruz), goat anti-FoxA2 (1:200, santaCruz), rabbit anti-Lmx1a (1:2000, M German), goat anti-Oct4 (1:200, santaCruz), rabbit anti-Sox2 (1:200, chemicon). Cells were washed 3 times in PBS followed by incubation for 1–2 hours with flourescence-labeled secondary antibodies (1:200, Jackson lab) and DAPI (1:1000).

### Quantifications and statistical analysis

The number of positively stained cells was quantified by counting 18 randomly selected fields per well corresponding to more than 1000 neurons in total. Similarly, the number of TH expressing cells that also co-expressed another marker was quantified by counting the total number of TH expressing cells and the proportion of these cells that also express another marker (Nurr1, Pitx3, En1). 3 wells per experiment were counted and the analysis was repeated 3–4 times for each condition. Analysis of variance (ANOVA) was used to compare means between groups. Differences between groups were determined using the Bonferroni Dunn test, with P-values less than 0.05 considered statistically significant.

## Authors' contributions

ML conceived the study and MP executed the experiments. Both authors contributed the experimental design, data interpretation and the writing of the manuscript.

## Supplementary Material

Additional file 1 **Supplemental Figure 1**. Quality control of FACS purification. PA6 and monolayer-derived *Sox1*-GFP expressing neural progenitors were FACS sorted and examined for the expression of undifferentiated ES cell (Oct4 and Sox2) and neural progenitor markers (Sox2). The majority of cells in the GFP^pos ^population expressed Sox2 but not Oct4. The percentage of Oct4/Sox2 positive cells was determined by dividing the total number of antibody stained cells against the number of DAPI nuclei. The top two rows were PA6-derived FACS purified neural cells whilst the bottom two rows were monolayer derived. Click here for file
