# Supplementary figures and images for: Early specification of dopaminergic phenotype during ES cell differentiation
Source: BMC Dev Biol. 2007 Jul 18;7:86. doi: 10.1186/1471-213X-7-86 (PMC1978208; doi:10.1186/1471-213X-7-86)

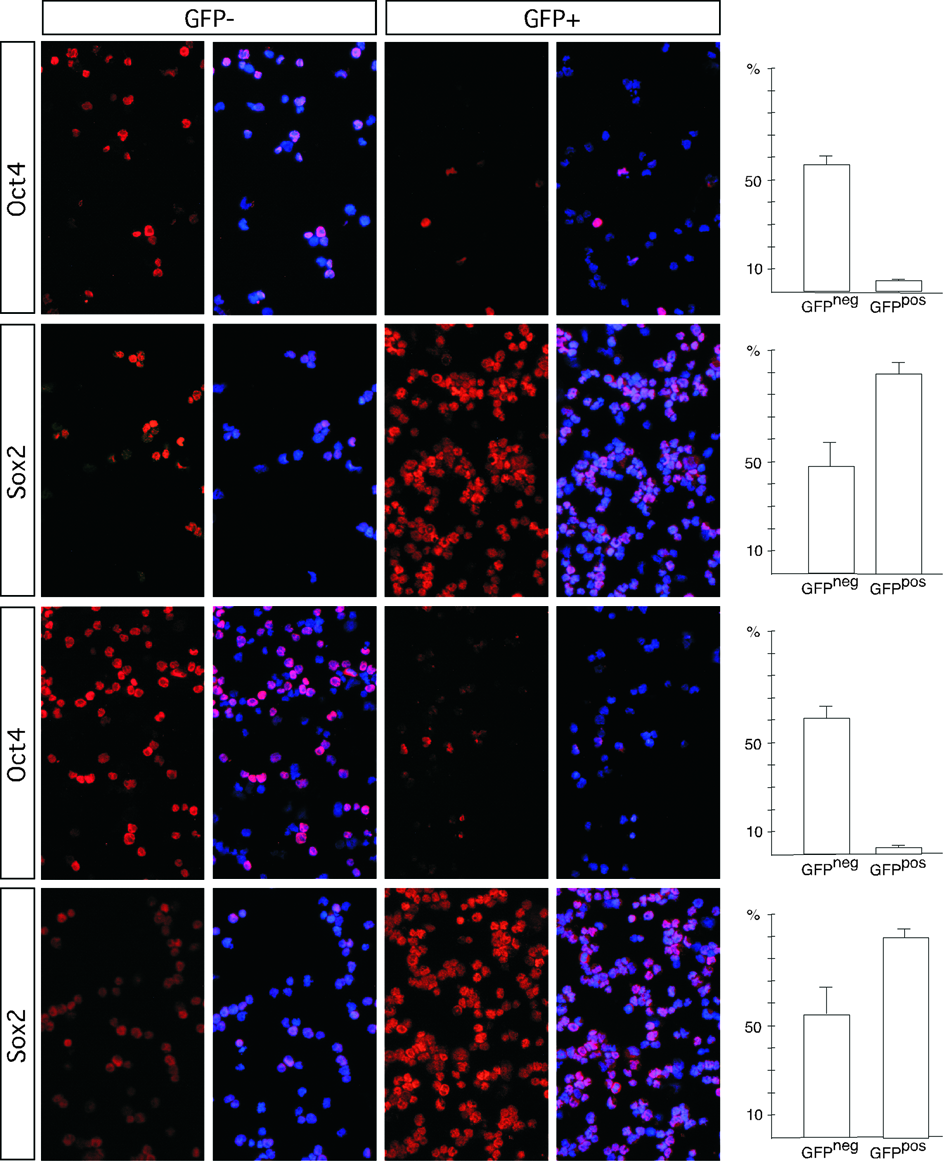

Supplement: Additional file 1 — Supplemental Figure 1. Quality control of FACS purification. PA6 and monolayer-derived Sox1-GFP expressing neural progenitors were FACS sorted and examined for the expression of undifferentiated ES cell (Oct4 and Sox2) and neural progenitor markers (Sox2). The majority of cells in the GFPpos population expressed Sox2 but not Oct4. The percentage of Oct4/Sox2 positive cells was determined by dividing the total number of antibody stained cells against the number of DAPI nuclei. The top two rows were PA6-derived FACS purified neural cells whilst the bottom two rows were monolayer derived. [file 1471-213X-7-86-S1.tiff]
